# Impact of the Safe Childbirth Checklist on health worker childbirth practices in Luapula province of Zambia: a pre-post study

**DOI:** 10.1186/s12889-018-5813-y

**Published:** 2018-07-18

**Authors:** Sandra Mudhune, Sydney Chauwa Phiri, Marta R. Prescott, Elizabeth A. McCarthy, Aaron Banda, Prudence Haimbe, Francis Dien Mwansa, Angel Mwiche, Francis Bwalya, Micheck Kabamba, Hilda Shakwelele, Margaret L. Prust

**Affiliations:** 1 Clinton Health Access Initiative, PO Box 51071, Ridgeway, Lusaka, Zambia; 2 Clinton Health Access Initiative, Boston, USA; 3 Ministry of Health, Lusaka, Zambia

**Keywords:** Maternal health, Newborn health, WHO Safe Childbirth Checklist

## Abstract

**Background:**

A strong evidence base exists regarding routine and emergency services that can effectively prevent or reduce maternal and new-born mortality. However, even when skilled providers care for women in labour, many of the recommended services are not provided, despite being available. Barriers to the provision of appropriate childbirth services may include lack of availability of supplies, limited health worker knowledge and confidence, or inadequate time. The WHO Safe Childbirth Checklist (SCC) includes reminders for evidenced-based practices at specific points in the childbirth process. Zambia is currently considering nation-wide adoption of the SCC, but there is a need for context-specific evidence. Beginning in September 2017, a program is being implemented in Nchelenge District to pilot use of the SCC, along with coaching that focuses on strengthening the systems that allow the essential practices in childbirth to be performed.

**Methods:**

This study will use a pre-post study design to measure health worker adherence to the essential practices for delivery care outlined in the SCC. Data will be collected through observations of health workers as they care for mothers during childbirth at four facilities. Data collection will take place before the start of the intervention, at 3 months, and at 6 months post-intervention. The primary outcome interest is the change in the average proportion of essential childbirth practices completed. A health worker questionnaire will be administered at the time that the SCC is introduced and 6 months later to gather their perspectives on incorporating the SCC into clinical practice in Zambia.

**Discussion:**

Findings are expected to inform plans for introducing the SCC in Zambia. This evaluation will aim to understand uptake and impact of the SCC and associated coaching in the context of a basic level of mentorship that the government could feasibly provide at a national scale.

**Trial registration:**

Clinical Trials.gov (NCT03263182) Registered August 28, 2017.

## Background

Despite some recent improvements, Zambia continues to experience high maternal and neonatal mortality rates. As of 2014, the maternal mortality ratio was estimated to be 398 per 100,000 live births [1], far above the average in developing countries of 239 deaths per 100,000 [2], while the neonatal mortality rate for 2009 to 2013 was 24 deaths per 1000 live births [1].

Maternal and new-born mortality can be prevented through the provision of basic health services for all women and appropriate emergency obstetric and new-born care (EmONC) services for women and infants who need it [3]. Although evidence suggests that basic services during pregnancy, at the time of delivery, and post-delivery, can prevent maternal and new born deaths, there are a number of barriers that prevent provision of basic maternity services. For instance, a critical challenge in Zambia and many other countries is that not all women deliver at a health facility and therefore do not have access to skilled health workers at the time of delivery [1]. A further challenge is that even when mothers go to health facilities, the appropriate staffing and supplies may not always be available to support the provision of essential childbirth services. Specifically, the facility must be staffed by a skilled birth attendant (SBA), which is an accredited health professional such as a midwife, clinical officer, doctor, or other health worker who has been educated and trained to proficiency in the skills needed to manage normal (uncomplicated) pregnancies, childbirth and the immediate postnatal period, and in identification, management and referral of complications in women and new-borns [4]. An SBA not only needs specific training to be able to provide the EmONC signal functions, but they should also have access to equipment, drugs, supplies, a referral system, and functioning communication and transportation infrastructure. Zambia’s health worker shortages are most acute in rural and remote areas with an estimated 12.4 clinicians per 10,000 persons as compared to 18.7 per 10,000 persons living in urban areas [5]. Both these ratios fall below the World Health Organization’s recommended threshold of 22.8 clinicians per 10,000 [6]. Even when mothers attend facilities for deliveries and the appropriate staffing and supplies are available, there remains a final barrier in assuring that the appropriate services are actually provided in a high-quality and comprehensive way. Possible factors associated with poor quality services could be limited health worker knowledge, confidence, and time.

The Safe Childbirth Checklist (SCC), developed by the World Health Organisation (WHO), is one tool that may help to improve the quality and safety of delivery services. The WHO SCC is based on the experience of the Safe Surgical Checklist, which demonstrated significant reductions in surgical complications and deaths [7]. The SCC consists of reminders to prompt evidenced-based practices that are essential to providing quality care at the time of labour and delivery. The tool is designed for use by birth attendants at four critical “pause points” in the delivery process: (1) at admission, (2) just prior to delivery, (3) in the immediate post-partum period, and (4) prior to discharge. A number of published studies have examined the process and effectiveness of introducing the SCC in specific country contexts, including India [8–13], Sri Lanka [14], Italy [15], Iran [16], and Namibia [17, 18]. A pilot study in India found that the delivery of evidence-based essential birth practices at each birth event increased from an average of 10 out of 29 practices prior to introduction of the SCC to an average of 25 out of 29 practices after the SCC had been introduced. However, there was no overall effect on mortality [19].

While the SCC has been implemented in sub-Saharan countries including Namibia and Uganda, the SCC has not previously been implemented in Zambia where maternal mortality is higher [20, 21]. The Ministry of Health (MOH) is currently considering nation-wide adoption of the SCC, but there is a need for context-specific evidence, specifically evidence about how to successfully implement the SCC, to generate lessons about the roll-out process in Zambia. This study will be unique compared to many previous studies that have investigated SCC adoption because the adoption of the tool will be integrated into a scalable, national framework for mentorship to health facilities.

## Objectives

Can the introduction of the Safe Childbirth Checklist and associated mentorship improve the adherence of skilled birth personnel to the essential practices of childbirth delivery?

The key outcome to be measured is the average proportion of essential childbirth practices completed.

## Methods

### Trial design

This is a pre-post study design that will include observing consecutive deliveries carried out in four selected health facilities before and after the introduction of the SCC. The study protocol was registered in the Clinical Trials.gov, a resource provided by the U.S. National Library of Medicine (NCT03263182).

### Study setting

The WHO SCC will be introduced in eight purposively selected health facilities in Nchelenge District, Luapula Province. Nchelenge District was chosen by the MOH to be the location for this pilot based on need. The eight facilities were selected based on criteria including: high demand for services, perceived need for support for quality improvement, and presence of a SBA to mentor. However, data collection for this evaluation will take place in only four facilities with high volumes for the observations to occur. The four sites include three health centers and one general hospital.

### Eligibility criteria

For facility observations, all consecutive deliveries will be observed. If a pregnant woman or health worker declines to participate in the consent process, no observations will be recorded.

### Interventions

The SCC will be piloted as part of a broader program to improve delivery of routine care for all women through a systems improvement approach focusing on health worker knowledge and skills; availability of supplies and equipment; ensuring good documentation and data use; and creating an enabling environment at health facilities in Zambia. This broader program is taking place in 14 districts in Zambia and aims to increase the availability, accessibility, and quality of maternal and newborn health services. While this program will be operating in 14 districts (and is being evaluated separately), the introduction of the SCC as a part of this package will be piloted in one district. The SCC intervention will involve several key steps and components:

- Adaptation of the WHO designed SCC tool to Zambia to produce a tool that is fully relevant to the Zambian context.
- Development of coaching tools to capture performance and track progress at individual and facility level.
- Training of coaches and district team to the adapted WHO SCC and coaching tools.
- Facility launches of the WHO SCC at all the eight target facilities to ensure facility staff understand the intervention.
- Coaching and supporting facility-level health workers on using the SCC by directly observing SBAs conduct deliveries as well as review of records for deliveries happening in the absence of the coach.
- Based on the data generated from the coaching tools, generate dashboards to be reviewed with coaches, facility staff and district teams so as determine challenges and identify issues of escalation within the health care system

### Outcomes

The primary outcome variable of interest is the change in the average proportion of essential childbirth practices completed per birth out of those observed from the list of selected tasks. We have developed a list of selected tasks that represent a sub-set of items in the SCC which are tasks that can be visually observed and that are non-conditional (in other words they should be performed on all mother-infant pairs regardless of health status). The list of selected tasks includes the following 21 items:

1. Was vaginal exam performed to check dilation?
2. Were sterile gloves used for the vaginal exam?
3. Was partograph started?
4. Did the health worker review danger signs with the mother (and companion)?
5. Were water and soap used to clean hands alcohol rub used on the mother just before delivery?
6. Were sterile gloves used at the time of delivery?
7. Were all of the following supplies available at the bedside? (Clean scissors or blade, Clean cord tie or clamp, Aspiration bulb or mucus extractor, Neonatal bag and mask, Oxytocin 10 units in syringe)
8. Did the primary health worker managing the delivery communicate with a health worker assistant to say that the birth will happen shortly and ask for assistance?
9. Was oxytocin administered?
10. Was newborn weight taken?
11. Was newborn temperature taken within 1 h after birth?
12. Was baby placed skin-to-skin on mother’s abdomen?
13. Was breastfeeding initiated?
14. Was maternal temperature obtained at admission?
15. Was maternal blood pressure obtained at admission?
16. Was maternal blood pressure obtained at discharge?
17. Was maternal temperature obtained at discharge?
18. Was newborn temperature taken at discharge?
19. Did health worker confirm that infant is feeding well?
20. Did health worker discuss family planning options with mother?
21. Did health worker plan a follow-up visit for mother and newborn and discuss danger signs?

### Study timeline

**Table:** 

	**M1**	**M2**	**M3**	**M4**	**M5**	**M6**	**M7**	**M8**	**M9**	**M10**	
**Hire and train data collectors**	X										
**Baseline data collection**	X										
**Launch of intervention**			X	X							
**Midline data collection**							X	X			
**End line data collection**										X	
**Data analysis**											X

### Sample size

We calculated the expected power that this observation period will give us to detect changes in practices based on data about delivery volumes at the four sites, using the following formula [22]:

$$ c=1+{\left({z}_{\alpha /2}+{z}_{\beta}\right)}^2\ast \frac{\left[\left({s}_0^2+{s}_1^2\right)\ast \left(1+\left(n-1\right)\rho \right)\right]}{n{\left({m}_0-{m}_1\right)}^2} $$

Due to the small number of facilities the equation utilizes the difference in the main proportion assuming a t-test will be utilized [23]. Where: c = number of facilities needed, Z_a/2_ and Z_B_ = type I (1.96) and type II error (0.84) respectively, s_0_ and s_1_ are the standard error at baseline (17%), ρ = interclass correlation (0.25). We estimate that at baseline 34% (m_0_) of the 15 SCC items will be observed per birth, based on previous work in India indicating that an average of 9.8 out of 29 items were observed per birth at baseline [12]. According to the Health Management Information System data from January to October 2016, there was an average of 390 deliveries per month across the four study facilities. This means that if we observed all deliveries for approximately 48 h in each site, we could expect to observe an average of 6.5 (n) deliveries per site. With such a sample size, the calculations indicate that we could detect an absolute increase of 24% in the proportion of adherence to SCC essential best practices (m_1_ = 58%).

For the secondary outcomes related to health workers characteristics and feedback, all health workers in the four study sites will be invited to participate as a convenient sample.

### Data collection methods

#### Delivery observations

Data will be collected through observation of health worker practices at baseline, midline and end line. Observations will be conducted by a team of four observers with clinical expertise so that they will be knowledgeable about clinical issues and capable of providing accurate assessments of whether SCC practices are completed or not. Observations will be conducted for a minimum of 3 days in all facilities, or as long as required to observe at least seven births.

It may not be possible for the observer to document every delivery process at all times, particularly if multiple deliveries are taking place at one time, so observations will relate to activities at four specific periods (pause points). As a part of the training process, the observing data collectors will observe births in parallel with a trainer or supervisor to ensure accuracy and consistency in recording the necessary information. Data collectors will be trained to recognize each of the practices on the checklist according to the definitions adapted from previous work [8]. Observation data will be recorded on standardized data sheets over the course of the delivery process. Data will be collected on paper forms and later entered electronically on portable touch-screen tablets that each observer will be equipped with.

#### Health worker questionnaires

A self-administered questionnaire [14] will be administered to health workers at the end of the facility-based SCC training and after all end line observations are completed to understand the overall success and acceptability of the tool and supportive activities. The questionnaires will collect basic information about the participant, their feedback on the process of using the SCC, and the appropriateness of the SCC for Zambia.

### Statistical methods

For the primary outcome, data from each delivery will be considered as a percentage of the 21 SCC items that were practiced, or in other words each pause point observation will be assigned a score from 0 to 100%. The differences over time will be examined using linear regression and accounting for clustering at the facility level as well as transformation to account for the bounded data. We will also consider clustering at the level of the primary provider responsible for overseeing the delivery. Sub-analysis will be conducted to account for the impact of availability of supplies on conduct of tasks. Within each facility in the sample, we will also compare the average score for all deliveries at baseline, midline and end line using a paired, weighted t-test to account for differences in the standard errors. Finally, to better explore meaningful change due to the program, we will examine if there is an increase in the proportion of births where the majority (75%) of tasks are completed; specifically, we will examine if the difference overtime in the average proportion of births where 75% of tasks were completed increases due to the intervention. To this end we will run individual-level logistic regression, accounting for clustering at the facility level.

For the health worker questionnaires, we will report descriptive statistics only, with baseline and end line comparisons.

### Study development and team

This evaluation is a partnership between the MOH of Zambia and the Clinton Health Access Initiative (CHAI) under the Demand-Driven Evaluations for Decisions (3DE) program. Since 2012, 3DE has been working with the MOH of Zambia to generate evidence in response to some of its most critical needs for evidence-based decision-making [23–27]. By using policy-relevant and rapid designs, 3DE evaluations have been able to improve the implementation of programs and policies.

The idea for this evaluation emerged out of requests from the MOH for evidence on practical ways to reduce maternal and newborn mortality. CHAI and the MOH were both aware of the SCC and reached out to Ariadne Labs for support in considering how the SCC could be brought to Zambia. The SCC introduction and coaching activities are being carried out by the Human Resources for Health program at CHAI, which aims to increase access to skilled attendance at birth, while the evaluation described in this paper is being carried out by 3DE.

## Discussion

Although intensive mentorship and guidance may indeed serve to improve adherence to the checklist, it is not feasible for such a program to be implemented at the national level. Instead, this process evaluation will aim to understand uptake of the SCC in the context of a basic level of mentorship that the government could feasibly provide at a national scale.

The outcomes, analysis, and recommendations of this evaluation are expected to inform plans for adopting the SCC in Zambia. Specifically, the results will help policy makers to understand the baseline quality of care as well as the potential for improvement by using the SCC. If only limited changes are observed, then national and international experts will collaborate to understand the reasons and propose further adjustments. Opportunities to combine the results with those from the broader process evaluation that will assess the scale-up of a national mentorship system will also be explored. If this intervention is scaled up across the country, support will be provided to the MOH in activities such as drafting an operational plan or providing high-level recommendations on how the intervention should be rolled out to achieve optimal results, based on the lessons learned from this evaluation.

## Abbreviations

EmONC: Emergency obstetric and new-born care
HCW: Health care worker
MOH: Ministry of Health
SBA: Skilled birth attendant
SCC: Safe Childbirth Checklist
TOT: Training of trainers
